# The complete chloroplast genome of an albino tea, *Camellia sinensis* cultivar ‘Baiye 1’

**DOI:** 10.1080/23802359.2019.1667889

**Published:** 2019-09-20

**Authors:** Wan-Jun Hao, Songlin Wang, Mingzhe Yao, Jian-Qiang Ma, Yan-Xia Xu, Liang Chen

**Affiliations:** Key Laboratory of Tea Biology and Resources Utilization, Ministry of Agriculture and Rural Affairs, Tea Research Institute of the Chinese Academy of Agricultural Sciences, Hangzhou, Zhejiang, China

**Keywords:** *Camellia sinensis*, chloroplast genome, Albino, genetic evolution

## Abstract

For obtaining the sequence and phylogenetic position of *Camellia sinensis* cultivar ‘Baiye1’, the complete chloroplast genome was determined. This chloroplast genome is 156,691 bp in length with overall GC content of 37.3%. It was comprised of a large single-copy (LSC) region of 86,585bp, a small single-copy (SSC) region of 18,276bp, and two inverted repeat (IR) regions of 26,083 bp. It contains 87 protein-coding, 8 rRNA, and 35 tRNA genes. Phylogenetic analysis showed ‘Baiye1’ and *C. sinensis* cv. ‘Longjing43’ were clustered into a group. These results may contribute to the further understanding of the albino phenotype and genetic evolution.

The tea plant [*Camellia sinensis* (L.) O. Kuntze] is cultivated worldwide for the production of nonalcoholic beverages. ‘Baiye1’ is a new tea cultivar, which is suitable for making famous and superior green tea. Its spring young shoots show albino at low temperatures. Previous studies showed that its albino phenotype is related to the amino acid content in young shoots (Li et al. 1999). In albino stage, the amino acid content can be up to two times that in ordinary green tea (Zeng and Liu 2015). Therefore, there is an important theoretical significance to unravel the underlying molecular mechanism of albino phenotype. In this study, we characterized the complete chloroplast genome sequence of ‘Baiye1’ as a resource for future genetic studies on albino and other related traits.

The young shoots were collected from ‘Baiye1’, growing in the China National Germplasm Hangzhou Tea Repository. A voucher specimen (GS00198) was deposited in the Herbarium of the Germplasm Resources Laboratory of the Tea Research Institute of the Chinese Academy of Agricultural Sciences. The total genomic DNA was used for constructing sequencing library (400 bp) and sequencing (pair-end 150 bp) was performed on an Illumina Hiseq 2500 platform (Illumina, San Diego, CA, USA). Approximately 6.70 GB of raw data was generated and filtered using NGS QC Toolkit_v2.3.3 (Patel and Jain 2012). The chloroplast genome was de novo assembled using NOVOPlasty (Dierckxsens et al. 2017) using a *C.sinensis rbcL* gene sequence (Genbank: MH270471.1) as seed and then manually corrected. The assembled chloroplast genome was annotated using DOGMA (Wyman et al. 2004).

The complete chloroplast genome of ‘Baiye1’ (Genbank: MN086819) is 156,691 bp in length, containing a large single copy (LSC) region of 86,246 bp, a small single-copy (SSC) region of 18,579 bp, and two inverted repeat (IR) regions of 26,083bp. The overall GC-content of the complete chloroplast genome is 37.3%, while the corresponding values of the LSC, SSC, and IR regions are 35.4, 30.0, and 43.0%, respectively. The chloroplast genome contained 87 protein-coding genes, 35 tRNA genes, and eight rRNA genes. Among all of these genes, four rRNA genes (i.e. 4.5S, 5S, 16S, and 23S rRNA), seven protein-coding genes (i.e. *ndhB, rpl2, rpl23, rps12, rps7, ycf15, ycf1, and ycf2*), and seven tRNA genes (i.e. *trnM, trnL, trnV, trnE, trnA, trnR, and trnN*) occur in double copies.

To confirm phylogenetic position of ‘Baiye1’, a molecular phylogenetic tree was constructed with the clustalw2.1 and MEGA4.0.2 based on eight complete chloroplast genomes of different cultivars and species from Theaceae genus (Figure 1). The result showed ‘Baiye1’ and *C. sinensis* cv. ‘Longjing43’ were clustered into a group. Two subspecies *C. sinensis* var. pubilimba and *C. sinensis* var. assamica and *C. sinensis* were clustered into a group. A good foundation for future genetic studies on albino and other related traits was laid through this chloroplast genome.

**Figure 1.**
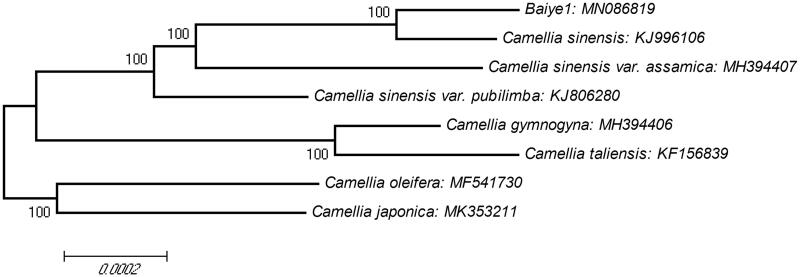
The phylogenetic tree based on 8 complete chloroplast genome sequences. The phylogenetic tree was constructed using neighbour-joining (NJ) method with 10000 bootstrap replicates. The bootstrap values were labeled at each branch nodes.
